# “English Disease”: Historical Notes on Rickets, the Bone–Lung Link and Child Neglect Issues

**DOI:** 10.3390/nu8110722

**Published:** 2016-11-15

**Authors:** Mingyong Zhang, Fan Shen, Anna Petryk, Jingfeng Tang, Xingzhen Chen, Consolato Sergi

**Affiliations:** 1 Department of Orthopedics, Tianyou Hospital, Wuhan University of Science and Technology, Wuhan 430064, China; zhangmingyong-08@163.com; 2 Department of Laboratory Medicine and Pathology, University of Alberta, Edmonton, AB T6G 2B7, Canada; fshen@ualberta.ca; 3 Comprehensive Pediatric Bone Health Program, Div. Pediatric Endocrinology, University of Minnesota, Minneapolis, MN 55455, USA; petry005@umn.edu; 4 Membrane Protein Disease and Cancer Research Centre, Provincial Innovation Center, Hubei University of Technology, Wuhan 430068, China; jingfeng@whu.edu.cn (J.T.); xzchen@ualberta.ca (X.C.); 5 Department of Physiology, University of Alberta, Edmonton, AB T6G 2R3, Canada; 6 Stollery Children’s Hospital, University of Alberta Hospital, Edmonton, AB T6G 2B7, Canada

**Keywords:** Vitamin D, rickets, history, histology, public health

## Abstract

Nutritional or classical rickets (here labeled as “rickets”) is a worldwide disease involving mostly infants and young children having inadequate sunlight exposure, often associated with a low dietary intake of Vitamin D. Rickets targets all layers of society independently of economic status with historical information spanning more than two millennia. Vitamin D is critical for the absorption of calcium and prevention of rickets in children as well as osteomalacia in adults. The initial and misleading paradigm of the 19th and 20th centuries that rickets may have been the consequence of infection has been, indeed, reversed following the identification of the Vitamin D molecule’s important role in the function of the immune system. Although traditionally considered limited to osteopathology, Vitamin D deficiency is now known to be linked to infection, inflammation, and carcinogenesis. In this review, we consider the key historical (Whistler, pre-Whistler and post-Whistler descriptors) and social facts around rickets; highlight the osteo-pathological features of rickets and the pathology of the upper and lower respiratory tract, stressing the fact that lungs remain the main secondary organ affected by Vitamin D deficiency; and emphasize the public health role in identifying the cases of child neglect or abuse based on the evaluation of the costochondral region.

## 1. Introduction

Rickets is no longer considered a disease of the past or a disease that is limited to low-income countries. In fact, there has been a resurgence of interest in rickets and Vitamin D status to the degree that was not thought of at the end of the 20th century. The number of publications and diseases that may be associated with Vitamin D deficiency is rapidly increasing, and more and more clinical laboratories are being asked to measure 25-hydroxy(OH)-Vitamin D (25[OH]D) levels [1,2,3,4,5] by both physicians and legal courts [6,7]. Large population surveys, such as the Nutrition Examination Survey and National Health, have suggested that many children and pregnant women may be affected by Vitamin D deficiency at some point during their lifetime [8,9,10,11,12,13]. Change of habits, such as less sunlight exposure, indoor living, dietary choices, and increasing rates of dietary allergy, are likely playing a significant role in the increase of this rate in some segments of the worldwide population according to the most recent evaluation of public health indicators [11,14,15,16,17,18,19,20,21,22]. 

Vitamin D is a fat-soluble vitamin, which has a unique metabolic pathway in that it is chiefly produced in the skin during sunlight exposure, unlike vitamins A, E, and K that are strictly absorbed from the diet. Conventionally, Vitamin D plays a leading role in calcium homeostasis. Vitamin D deficiency is a major cause of rickets, osteomalacia, and osteoporosis in the human population [23,24,25,26]. More recently, the link between Vitamin D deficiency and impaired immunity, inflammatory response and dysregulated cancer pathways has been emphasized [27,28,29]. In fact, immune system cellular components possess Vitamin D receptors (VDRs) that can bind the active form of Vitamin D (calcitriol, 1,25-dihydroxyVitamin D, 1,25(OH)_2_D) [30,31,32,33]. The storage form of Vitamin D, which is 25-hydroxyvitamin D, or 25(OH)D, can be converted by activated T and B cells to 1,25(OH)_2_D in human cells in vitro [34]. The 1,25(OH)_2_D acts locally on the immune cells in both autocrine and paracrine manners. Moreover, peripheral blood mononuclear cells (PBMC) harbor VDRs providing support for a significant role of Vitamin D in the regulation of the immune system and infectious diseases [35]. Box 1 includes a list of etiologic factors associated with Vitamin D deficiency.

In this review, we report on the history of the “English Disease”, highlight the osteopathological features of rickets as well as the pathology of the respiratory tract, and discuss the leading role of public health in identifying cases of child neglect or abuse, and the impact of socio-ideological debates.

Box 1 **Etiologic Factors involved in Vitamin D Deficiency.**

**↓ Nutritional intake of Vitamin D**

- Exclusive breastfeeding > 6 months
- ↓ Maternal Vitamin D stores
- Malabsorption
  ○ Gluten-sensitive enteropathy (GSE)
  ○ Non-GSE malabsorption syndromes
  ○ Cystic fibrosis (CF)
  ○ Non-CF-related pancreatic insufficiency
  ○ Biliary atresia (BA)
  ○ Non-BA extrahepatic obstructive cholangiopathies

**↓ Synthesis or ↑ degradation of 25(OH)D**

- Liver cirrhosis
- Non-cirrhotic chronic liver disease
- ↑ Vitamin D metabolism (e.g., isoniazid, rifampicin, and anticonvulsants therapy)
- ↑ Skin pigmentation
- ↓ Sun exposure:
  ○ Sunscreen with protection factor > 8, type of clothing, shades
  ○ Latitude > 40 degrees (North or South), “long winters”, air polluted geographical areas, and “perennial clouding” geographical areas

## 2. Historical Notes

The year 1645 is commonly recognized as the opening year for the scientific literature on rickets. That year is mostly famous for several events linked to the English Civil War and to the battle of Jankau of the Thirty Years’ War. In the same year, two public health events happened, i.e., Black Death (plague) causing many town or city councils, such as Edinburgh, to prohibit all gatherings except for weddings or funerals; and Jeanne Mance founding the Hôtel-Dieu de Montréal, the first hospital in North America [36]. In 1645, David Whistler (1619–1684) defended his M.D. thesis at the University of Leiden, the Netherlands, with the title “*De morbo puerile anglorum, quem patrio idiomate indigenae vocant the Rickets*” (Concerning the disease of English children, which in English it is called “Rickets”) [37]. History of medicine is fascinating and digging into the archives may reveal impressive results. To the best of our knowledge and following retrieval of documents, David Whistler is known as the first physician who published an imposing scientific work on rickets. The English disease or “*Morbum Anglorum*” was more than a disease and had and will have notable impact on social and industrial life. Dr. Whistler named the rickets “*Paedosplanchosteocaces*”, a rational, but probably awkward name, which was not favored by the scientific community and future generations [38]. In 1684, Dr. Whistler was appointed as President of the College of Physicians in England, but he died the same year when his thesis was re-published. 

The awareness of the disease must have existed years before Dr. Whistler’s thesis, as is evident in the painting by Hans Burgkmair completed in 1509 [39]. Neither poor nor powerful families were able to prevent their children from suffering from rickets during the Renaissance. Even members of the Medici family, one of the most powerful families during the Italian Renaissance (16th and 17th centuries), suffered from Vitamin D deficiency-related bone changes, as shown by examination of their skeletons [40,41,42,43]. Rickets was also known in Italy in the 16th century based on the description of a child of the Medici family who was found to suffer from rickets [41]. A child of the Medici family identified as Don Filippino (1577–1582), son of the Grand Duke Francesco I (1541–1587) and Johanna from Austria (1547–1578) was found still wearing high-social status clothes during the search of the Medici Chapels in the Basilica of San Lorenzo in Florence, Italy. Osteo-archeologists using ^13^C and ^15^N bone collagen analysis could identify a number of pathological lesions, including porosity shown in the skull, orbits, costochondral regions, and growth plates of long bones in addition to the enlargement of rib ends and bending of long bones [42]. Probably, prolonged breast-feeding, often for children older than two years, inadequate solid food diet, and poor sunlight exposure have been causative at that time. Children who were frail were often confined indoor with poor exposure to sunlight. Another remarkable pre-Whistler rickets case may have been represented in Caravaggio’s *Amore dormiente* [44,45,46].

Earlier pre-Whistler descriptors may be found in a Greek physician, Soranus (Σωρανός) of Ephesus, who practiced in Rome during the 1st and 2nd centuries as one of the chief representatives of the Methodic school of medicine. He describes some bone deformities, which may have been rickets, attributing this disease to improper childcare. Another reference may have been that of Claudius Galenus (Κλαύδιος Γαληνός) (of Pergamon, Mysia, Anatolia, now Bergama, Turkey), another Greek physician of the Roman Empire, who also wrote of a disease suggesting the appearance of rickets in *De Morborum Causis*. However, he attributed the chest deformities of the affected infants and toddlers to the pressure of swaddling clothes [47,48,49,50,51]. Pre-Whistler descriptions also include pre-Christian era episodes in China that help to delineate some difficulties and ambiguities of the clinical picture of rickets. Noel Joseph Terence Montgomery Needham CH FRS FBA (1900–1995) was a British scientist, historian and sinologist, who travelled multiple times into Eastern countries [52]. Indeed, a number of diseases, including rickets, were brilliantly known in ancient China. Iconography of the industrial cities of Germany and Holland, at least 200 years before Whistler’s description, definitely support the thesis that rickets existed to some extent at all times and periods of the world’s history [46,53,54].

It is common knowledge that Dr. Whistler’s first description of rickets was eclipsed by Dr. Glisson’s scientific contribution of five years later. Francis Glisson (1597–1677), born and raised in Rampisham, Dorset, published a book on rickets, which was particularly appreciated by both the scientific and public communities. His publication “*De Rachitide Sive Morbo Puerili, qui Vulgo The Rickets dicitur, Tractatus*” is dated 1650 [55]. Dr. Glisson received the credit, but in the text more contributing authors were mentioned as well. This report has been recognized as the first official statement from a medical college for both scientific and public audiences using physical evidence and inquiry rather than purely theoretical speculation, emphasizing both clinical and anatomical features of the disease with the help of the morbid anatomy. Glisson’s book “*De Rachidite*” (1650) was inspiring. Glisson, in addition to being *Regius* Professor of Physics, Cambridge (1636–1677), and President of the Royal College of Physicians (1667–1670), was also one of the first fellows of the Royal Society (1663). 

The existence of rickets at that time may be further supported by an autopsy performed on an 18-month-old child in June 1666 by John Locke. He mentioned in his report that the child was harboring ‘rachitic bony changes’ [56,57,58,59]. Rickets was identified as the cause of death, although the child also had an Ebstein anomaly of the heart, becoming the topic of fervent discussion [60,61,62]. John Locke FRS (1632–1704) was an English philosopher and physician, following the tradition of Sir Francis Bacon, supporting the social contract theory, with encyclopedic works spanning from epistemology and science to political philosophy [57]. 

Glisson’s influential position in medicine made rickets soon become the English disease of the pre-Industrial Revolution in the British Isles. Among several causes, dietary deficiencies, social, economic and environmental issues must have played a significant role, as cities and towns of Victorian England and Wessex were particularly enveloped in smog. Glasgow physician, William Macewan, had probably unsurpassed experience with children with rickets using osteotomy to treat the leg deformities at the end of the 19th century [63]. He suggested that environmental factors (e.g., sunlight deprivation) dominated the poor nutrition. Cod liver oil was often proposed as one of the therapies and Thomas Percival in Manchester in the 18th century was the first reporting its medical use [64]. However, many physicians were hypercritical against cod liver as a specific therapy for rickets [65]. After the 1st world war (WWI), the “sunshine movement” seemed to prevail, although the medical community and the public were of the opinion that both specific dietary deprivations and environmental restrictions contribute to rickets [28]. Two more prominent figures may have approached and conquered the rickets scene, i.e., Florence Nightingale and Hugh Owen Thomas. Ms. Nightingale, OM, RRC (1820–1910) was an English social reformer and professional statistician. She was particularly worried about *miasma*, a postulated malignant influence in the atmosphere, and characteristically formulated how *miasma* may cause and promote several diseases [66]. Mr. Thomas (1834–1891) was a Welsh surgeon and is considered the father of orthopedic surgery in Britain. His adoptive family was engaged in bone-setting, a practice of joint manipulation, forerunners of chiropractors, osteopaths, and physical therapists [67]. Thomas also devised beds for children improvised from soap boxes placed outside their homes, and later, on the sun-exposed balconies at the Sea Side Hospital [68]. The nurses and physicians became inspired by seeing a home for crippled children and, with the help and powerful influence of Robert Jones, a hospital model took kindly shape involving location in the countryside, regular open air, and plenty of sunshine time. Although rickets was probably not the primary diagnosis of children admitted to the “sunshine” hospitals, the sunshine must have promoted healing of rickets in many patients. 

Undeniably, the “sunshine” movement was in full swing probably years before scientific proof became available in its support. Although the association between rickets and air pollution functioning as a barrier to sunlight in industrial and high density domestic areas of Britain using coal burning was not appreciated, and heavily contested by some layers of the public and the economic parties, the British Medical Association (BMA) was a pillar of change. In 1889, a BMA working party issued an official report on the geographical distribution of rickets as well as other diseases involving large industrial towns and their environments at that period and a Cumbrian general practitioner published a report, which became key for the treatment of rickets [69]. Following his graduation from Edinburgh University, Dr. Theobald Adrian Palm (1848–1928) joined a Medical Mission in Niigata, Japan. Dr. Palm was impressed by the absence of patients with rickets, which impelled him to request his colleagues as to their experiences in other countries worldwide. Dr. Palm’s data led to an inspiring epidemiological conclusion that there is an inverse relationship between prevalence of rickets and exposure to the sun [70,71]. Between 1914 and 1918, most of the Austro-Hungarian and Prussian-German empires were under a stiff blockade imposed by the British navy and Russian army. Inadequate food supply for most of the population was common and rickets was common in children and adolescents in wartime Vienna, Budapest, and Berlin [71]. Orphanages were not well organized and most of these poorly nourished orphans had only limited time of outdoor activity. In this tragic context, Kurt Huldschinsky (1883–1940), a German Pediatrician of Polish heritage (Prussian) noted the pale skin of his young patients. Dr. Huldschinsky served in the Reichswehr as a field medic during WWI. In the winter of 1918/1919, he successfully demonstrated how rickets could be treated with ultra-violet (UV) lamps [72]. It is estimated that an impressive proportion of German children, perhaps half of them, suffered from rickets. Heliotherapy was a common protocol for numerous illnesses and seemed to be promising in healing or at least relieving some manifestations of rickets. He tried to adapt existing X-ray technology to generate artificial UV and photographs of that time show radiation-exposed children wearing sunglasses. He gave children calcium supplements and irradiated them with quartz mercury-vapor lamps, which emit wavelengths ranging from 200 to 600 nm, ultraviolet B (UVB) being wavelengths between 290 and 320 nm [73,74,75]. Interestingly, Huldschinsky showed that exposure shined on only one arm indeed cured rickets in both arms, and hypothesized that a chemical compound was synthesized in response to UV light that could intimately diffuse throughout the affected child. 

Dr. Huldschinsk’s hypothesis was brilliant and came at the time numerous exciting publications came out following Casimir Funk’s discovery in 1912 of vital amines or vitamins [76]. Sir Edward Mellanby (1884–1955) discovered Vitamin D and its potential role in preventing rickets in 1919 [77,78,79]. Sir Mellanby uniquely discovered that cod liver oil could reverse rickets in porridge-fed caged dogs. The appointment as professor of pharmacology at the University of Sheffield in 1920 was followed by the secretary position he received at the Medical Research Council from 1933 to 1949 and was awarded the Royal Medal, the Buchanan Medal and knighted in 1937 having received the Knight Grand Cross of the Most Excellent Order of the British Empire (GBE) in 1948. His suggestions were confirmed by two independent and thorough pediatric studies in Vienna, Austria emphasizing that both cod liver oil and sunlight exposure were indeed successful in healing rickets [80]. Pre-Whistlerian, Whistler’s and post-Whistlerian data form the basis for the current recommendations of several medical societies worldwide [65].

## 3. Osteopathology

**Figure 1:**
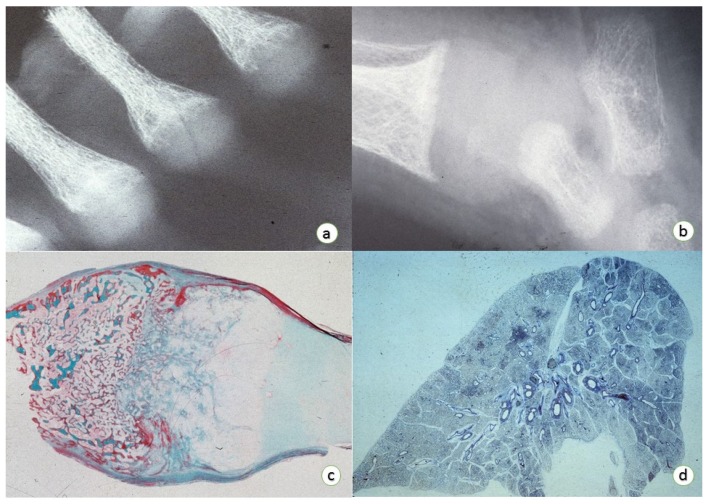
Rickets and pneumonia: (**a**) Roentgenogram of the anterior chest showing prominent costo-chondral junctions. The anterior chest was cut to obtain tissue for plastic and paraffin embedding. (**b**) Roentgenogram of the right ankle showing wide and concave distal end of tibia. The distance from the distal end of the tibia to the tarsal bones is increased due to the large “rachitic metaphysis”, which is not calcified and does not appear on the roentgenogram. The radiographic change corresponds to the palpable thickening of the ankles on physical examination. (**c**) Histology of the right femur showing the expansion of the growth plate identified at the 6th costo-chondral region (×2.5 original magnification, Masson’s trichromic stain). (**d**) Whole-mount of the lung of an infant with rickets showing lobar pneumonia (personal archived material, C. Sergi).

Vitamin D deficiency results in rickets in children with growing bones, and osteomalacia in adults with completed growth and closed growth plates [7]. The characteristic features of rickets are centered during the endochondral ossification, which is responsible for the longitudinal growth of long bones. In endochondral ossification proliferating cartilage is progressively replaced by bone. A good vascular supply is crucial for this process. The growth plates or epiphyseal plates are the locations where the endochondral ossification occurs. No further longitudinal growth takes place once the growth plates are closed. The appositional or intramembranous ossification also contributes to the growth of long bones, but mostly in width and occurs mainly along periosteal surfaces in flat bones and long bones. There is no intermediate cartilaginous phase in the appositional ossification. It begins with proliferation of mesenchymal connective cells that build up a connective tissue based membrane, which subsequently is replaced directly by mature bone. Two types of bone are seen according to the stage of maturation, woven bone or lamellar bone. The first is immature bone present during fetal development or in the early stages of bone repair. The collagen fibers are randomly distributed. A crisscross pattern of the collagen fibers is recognized in the immature bone on light microscopy. Subsequently, the tissue is mature bone, which is present in normal adult stages and a light microscopic view shows collagen fibers that are arranged in a parallel fashion. Children with rickets have irregular, broadened, cup shaped epiphyseal growth plates around knees, ankles and wrists, while adults with osteomalacia have bone formed during remodeling, which is poorly mineralized, predisposing them to fractures. Clinico-radiological and biochemical evaluations are usually sufficient for diagnosis. Radiologically, there is a generalized osteopenia with multiple bilateral and symmetrical linear fractures, which are interpreted as insufficiency or stress fractures. Histology of the growth plate shows thickened, poorly defined architecture. There is, particularly on metaphyseal side, a disarrangement of the growth plate with prolonged structures of uncalcified cartilage potentially extending into metaphysis and wide osteoid seams (Figure 1). 

In adults, there is a wide, non-calcified matrix surrounding disorganized bony trabeculae associated with irregular and granular junction between osteoid and mineralized bone. Bone volume may also be increased. In Box 2, the characteristics of the epiphyseal physis and medullary bone are highlighted [7]. In particular, the zone of provisional calcification seen in children with rickets is poorly defined with only a few of the defective bars between the almost non-existent columns of chondrocytes showing deposition of mineral. Two diagnoses need to be ruled out clinically, biochemically and/or genetically: hypophosphatasia [81,82] and metaphyseal chondrodysplasia [67].

Box 2 **Epiphyseal Physis and Medullary Bone Histologic Changes in Rickets.**

**Epiphyseal Physis:**

(1) Enlargement of the epiphyseal plate in height and width.
(2) Enlargement and distortion of the hypertrophic cell zone with poorly definition of the zone of provisional calcification associated with loss of the normal architecture of the cellular columns with small amount of intervening matrix.
(3) Failure of adequate mineralization and vascular invasion.
(4) Normal resting cartilage with prolongations (so-called “tongues”) of viable cartilage without histologic evidence of active endochondral replacement.

**Medullary Bone:**

(1) ↓ Medullary bone with thin and irregular bony trabeculae with an extended layer of un-mineralized bone (osteoid seams) surrounding mineralized bony segments.
(2) ↓ Bony component in both trabecular and cortical bones.

## 4. Respiratory Tract Pathology

In the last two decades, an intense investigation around Vitamin D and its metabolism has been recorded identifying its important roles in cell signaling during both the adaptive and innate immune response to bacterial and viral infections [30,31,32,83,84,85,86,87,88,89,90]; for example, cholecalciferol in the management of tuberculosis (TB) [14,91,92]. In addition to the conventional T, B, and natural killer (NK) cells, there is a group of recently recognized innate lymphoid cells (ILCs) involved in mediating the host defense [93]. Unlike T and B cells, which are active in adaptive immunity against pathogenic microorganisms in an antigen-specific fashion, ILCs respond to invaders in the absence of somatically rearranged antigen receptors and in a prompt manner. Human respiratory cells are able to convert 25(OH)D to 1,25(OH)_2_D in human respiratory epithelium cells in vitro [94]. Moreover, several types of respiratory infections, including respiratory syncytial virus and tuberculosis have been associated with 1,25(OH)_2_D and/or 25(OH)D in the immune response in vitro [94,95,96]. There is a plethora of articles identifying the link between 25(OH)D or 1,25(OH)_2_D deficiency and infectious diseases cited in Sundaram and Coleman (2012) [27]. Observational studies have been performed with influenza virus [97,98,99,100,101], TB [102], RSV [95,103], and other respiratory diseases [104,105,106,107]. 

The National Health and Nutrition Examination Survey (NHANES), a program intended to measure the health and nutritional status of both children and adults in the United States uniquely combining interviews and physical examinations, has issued estimates that about half of the U.S. population have 25(OH)D lower than 30 ng/mL [106]. More severe Vitamin D deficiency, as defined as 25(OH)D lower than 10 ng/mL, may be a condition affecting 2% of Americans [106]. In fact, significantly lower levels of Vitamin D seem to have been present in children with respiratory diseases, older adults, women, and individuals that have dark skin pigmentation, which may be an at-risk population [108,109,110,111]. 

There is a series of studies cited in Sundaram and Coleman (2012) [27] showing lower levels of 25(OH)D in serum to be associated with an increased risk of respiratory infections in infants, children, and adults. In two large, population-based investigations carried out in the United Kingdom [104] and the United States [112], there was evidence of a strong, dose-responsive association between lower levels of 25(OH)D and increased risk of upper respiratory infections. In particular, children appear to be more susceptible to increased severity of acute respiratory infection, specifically lobar pneumonia, if they have a concurrent Vitamin D deficiency [27]. The Vitamin D seems to act as powerful immune system modulator, preventing excessive expression of inflammatory cytokines and increasing the “oxidative burst” potential of activated macrophages. This vitamin stimulates the expression of potent antimicrobial peptides, present in neutrophilic granulocytes, monocytes, natural killer cells, and in bronchial/alveolar cells of respiratory tract, playing a major role in protecting the lungs from infection [27]. The detailed mechanisms are under intense investigations, but there is some evidence that certain single nucleotide polymorphisms (SNPs) of Vitamin D receptor (VDR), are associated with the risk of acute infections of the lower respiratory tract in children [113]. The TaqI polymorphism has been associated with lower VDR protein levels and the FokIff SNP seems to cause some downregulation of the Vitamin D target gene, CYP24A1, which codes for an enzyme that degrades 1,25(OH)_2_D [114,115]. Thus, more studies on SNPs and VDR functionality may be important in the future.

Pneumonia remains, indeed, the most important cause of morbidity and mortality in children less than five years old worldwide [7]. About 43% of 200 Iranian children admitted to the hospital with radiographically proven rickets had classic signs of bronchopneumonia [116], about half of 250 Kuwaiti children with rickets had pneumonia or bronchopneumonia [117], and even four out of five of Egyptian children with rickets showed acute respiratory infections at some point during hospitalization [118]. As mentioned earlier, this association between rickets and pneumonia may be explained by the effects of 1,25(OH)_2_D_3_ on the immune system, because calcitriol stimulates phagocyte-dependent and antibody-dependent macrophage cytotoxicity modulating simultaneously T and B cell function [30,31,32,83,84,85,86,87,88,89,90]. 

Supplementation with Vitamin D in randomized controlled trials has produced variable results [27]. In fact, the supplementation of calcitriol (1,25(OH)_2_D_3_) as a compound or adjuvant molecule to increase the efficacy of an influenza vaccine in both general adult population and human immunodeficiency virus-infected adults has shown no significant effect [119,120,121]. A study involving Vitamin D supplementation to patients harboring prostate cancer was also associated with no improved serologic response to an influenza vaccine. However, the baseline concentration of Vitamin D in the same cohort of patients seems to have been associated with an enhanced response to influenza vaccine inoculation [100]. Japanese school children receiving Vitamin D supplementation also experienced a significant reduction of seasonal influenza A [99] with some partial support from another investigation [101]. In a systematic review, 13 controlled trials were considered [122]. Yamshchikov et al. suggested that further research into Vitamin D supplementation may be needed for tuberculosis and viral infections of the upper respiratory tract using a rigorously designed clinical trials methodology to thoroughly evaluate the relationship between Vitamin D status and the immune response to infection [122]. 

## 5. Public Health and Socio-Ideological Debate

Rickets is not only a public health issue, but also a topic of socio-ideological debate, particularly in England. The deficiency of Vitamin D persisted as a “familiar disease” of working class until the 2nd world war (WWII) when it was virtually eradicated through the institution of supplementation programs [123,124]. The new National Health Service (NHS) developed a number of preventions for disease prevention [125]. The success of Britain over rickets in wartime included first, rationing and state-run canteens with sufficient nutritious diet for poor layers of the society; second, the mandatory supplementation using exclusive milling of bulky, nutrient-rich high-extraction flour and fortifying flour with calcium, as well as margarine with Vitamin D and A; third, Vitamin D supplementation of the nursing mothers, infants and toddlers [126,127]. In 1954, victory over rickets was announced [123,128]. However, in the early 1960s, an apparent increase in rickets was observed [129,130]. In the early 1960s, higher rates of rickets were seen among recent migrants to Britain from the “New Commonwealth” (the so-called “Asian Rickets”) [123,124,131]. The health of Asian and non-white migrants became a delicate issue brought into political discussions steadily. As suggested by Bivins [123,124], the ideology and politics behind Vitamin D deficiency and/or fortification are intrinsically intertwined with the history of rickets and governmental choices. Medical professionals and the British Medical Association continued to insist on the return of rickets [123]. Rickets regained its place on the discussion agenda of the parliamentary debate only in 1971 when a further reduction in the delivery of free school milk was proposed because of the fragile economy. In the meantime, the arrival of refugee families, particularly of South Asian descent, filled tabloids and newspapers. The rise of the identity of rickets as “Asian Rickets” was gaining debate in the political sphere ignoring a generation of British-born children of Asian heritage. The reality is that the English Disease was only apparently transformed into the Asian disease, because osteomalacia was still prevalent among elderly of all ethnic origins. Similar to the 1970s and 1980s calls for fortification, wider supplementation and more active governmental engagement have produced little action even today, because rickets has not disappeared entirely from the British Isles.

Rickets is probably underdiagnosed in many Western countries, but also in Asia [132,133,134,135,136], and it is also rising in countries with fast changing nutritional habits, e.g., China [22,132,133,135,137,138,139,140,141,142,143,144,145,146,147,148,149,150]. Children in Western countries are, however, also at risk if living in some high-risk groups. Dwyer et al. (1979) studied records of the dietary intake of preschool vegetarian children and found that macrobiotic vegetarian diets provided marginal amounts of Vitamin D, calcium, and phosphorus [151]. Moreover, the same authors found that Vitamin D supplementation was rarely given. Children on macrobiotic diets showed physical and roentgenographic findings indicative of rickets more often than in the case of other vegetarian diets. In fact, Dagnelie et al. also studied the Vitamin D metabolism in Caucasian 10–20-month-old infants on a macrobiotic diet and on omnivorous diets. They found that low availability of calcium in the macrobiotic diet was an independent factor in causing the high prevalence of rickets in these infants [152]. Macrobiotic diet avoids milk products and includes a high fiber intake. Both components may have adverse effects on bone development in young children. Thus, rickets has been reported and is a dramatic reality in immigrant children not only in the United Kingdom, but worldwide [153,154,155,156,157,158,159]. 

Rickets due to child abuse and child neglect is unfortunately also present in our societies [6,7,16,160,161,162,163,164,165,166]. Child neglect rarely receives attention, usually only when traces of physical ill-treatment can be observed [162,164]. Infections of the lower respiratory tract may pass unnoticed by a social worker. Public health policies are not focused on rickets (Vitamin D supplementation and screening programs) and many pediatricians think that it is a disease of the past, assuming that children have adequate access to fortified milk. The latter, however, does not take into account either the ever-growing consumption of carbonated beverages instead of milk or the social situation of children, particularly among the refugees [167,168,169,170,171,172,173]. Alcohol abuse is present in many families that abuse or neglect children. Alcohol and other drug use can affect children both prenatally through maternal alcohol consumption and postnatally through environmental factors that reinforce alcohol intake and their own alcohol/drug abuse [174,175,176,177,178,179,180,181]. Child neglect may also be unintentional [175]. Other social risk factors could also play a role as trigger factors, such as immaturity, unemployment, and poverty.

Compulsory home-visitation service by nurses for improving maternal and child health is probably the best preventive measure. This has been confirmed by a 15-year follow-up study of a randomized trial in a semirural community in New York [182,183]. In an interesting evaluation of child neglect in Dusseldorf, Germany, Dr. Trube-Becker stresses that the suspicion of negligence was only raised by the autopsy findings and no doctor or social services have been called upon when the child was still alive, highlighting the importance of a strong cooperation between family doctors, social workers, health authorities, and pediatric clinics staff to identify and address neglect early on [162,164]. Educational programs for young parents need to be updated without criminalization of some sectors of the populations, and the learning process should be carefully evaluated in a close follow-up, particularly in families at risk. Doctors should not limit their services to the physical health of children, but also their social and emotional wellbeing to help identify the family problems and risk factors. The value of a valid program of home-visitation service by nurses and other healthcare providers should not be underestimated as a means to reduce the number of cases of child abuse and neglect.
